# Supplementary material for: Risk of Symptomatic Intracranial Hemorrhage After Mechanical Thrombectomy in Randomized Clinical Trials: A Systematic Review and Meta-Analysis
Source: Brain Sci. 2025 Jan 11;15(1):63. doi: 10.3390/brainsci15010063 (PMC11764228; doi:10.3390/brainsci15010063)
Supplement: Supplementary file 1 [file brainsci-15-00063-s001.zip › brainsci-3366188-supplementary.pdf]

Supp. Table 1. Search strategy and results of the different databases.

Database	Search strategy	Results
**PubMed**	((("stroke"[All Fields]) AND ("endovascular therapy"[All Fields] OR "thrombectomy"[All Fields])) AND ("sICH"[All Fields] OR "intracranial hemorrhage"[All Fields] OR "ICH"[All Fields] OR "hemorrhage"[All Fields])) AND (randomizedcontrolledtrial[Filter])	**130**
**Scopus**	(TITLE-ABS-KEY ("stroke") AND TITLE-ABS-KEY ("endovascular therapy" OR "thrombectomy") AND TITLE-ABS-KEY ("sICH" OR "intracranial hemorrhage" OR "ICH" OR "hemorrhage") AND TITLE-ABS-KEY ("randomized clinical trial" OR "randomized controlled trial" OR "randomized trial" OR "RCT" OR "controlled trial" OR "clinical trial"))	**1704**
**Web of Science**	TS=(("stroke") AND ("endovascular therapy" OR "thrombectomy") AND ("sICH" OR "ICH" OR "intracranial hemorrhage" OR "hemorrhage") AND ("randomized controlled trial" OR "randomized clinical trial" OR "RCT" OR "controlled trial" OR "clinical trial" OR "randomized trial"))	**393**
**Embase**	'stroke':ti,ab,kw AND ('endovascular therapy':ti,ab,kw OR 'thrombectomy':ti,ab,kw) AND ('sich':ti,ab,kw OR 'intracranial hemorrhage':ti,ab,kw OR 'ich':ti,ab,kw OR 'hemorrhage':ti,ab,kw) AND ([controlled clinical trial]/lim OR [randomized controlled trial]/lim)	**623**

Supp. Table 2. Definitions of Groups 1 and 2 of each trial together with sICH rates and definitions.

Trial	Group 1	Group 2	Group 1 Event	Group 1 Total	Group 2 Event	Group 2 Total	Timing (h)	sICH
DIRECT-MT	MT	MT+ IVT (alteplase)	14	327	20	329	<4.5	Heidelberg criteria
RESILIENT	MT + BMT	Best medical treatment (BMT)	5	11	5	111	<4.5	parenchymal hemorrhage type 2 associated with an increase of ≥4 points in the NIHSS score or leading to death (SITS-MOST)
THERAPY	MT + IVT	IVT (alteplase)	4	43	6	62	-	concomitant ≥4 point worsening in NIHSS as recorded by a blinded, NIHSS-certified assessor (SITS-MOST)
DAWN	MT + BMT	Best medical treatment (BMT)	6	107	3	99	6-24	parenchymal hemorrhage type 2 associated with an increase of ≥4 points in the NIHSS score or leading to death (SITS-MOST)
RESCUE-Japan LIMIT	MT + BMT	BMT	9	100	5	102	<6	parenchymal hemorrhage type 2 associated with an increase of ≥4 points in the NIHSS score or leading to death (SITS-MOST)
RESCUE BT	MT + Placebo	MT + Tirofiban	31	483	45	462	<24	Heidelberg criteria
CHOICE	MT + Placebo	MT+ IVT (alteplase)	2	52	0	61	<24	≥4-point increase on the NIHSS score (SITS-MOST)
SELECT2	MT + BMT	Best medical treatment (BMT)	1	178	2	174	<24	≥4-point increase on the NIHSS score (SITS-MOST)
SWIFT PRIME	MT + IVT (tPA)	IVT (tPA)	0	98	3	97	<6	-

SKIP	MT	MT + IVT	6	101	8	103	<4.5	National Institute of Neurological Disorders and Stroke (NINDS)and Safe Implementation of Thrombolysis in Stroke–Monitoring Study (SITS-MOST) criteria
ATTENTION	MT + BMT	BMT	12	226	0	114	<12	modified Safe Implementation of Thrombolysis in Stroke–Monitoring Study (SITS-MOST) criteria
DEVT	MT	MT + IVT (alteplase)	4	115	5	117	<4.5	Heidelberg classification
DIRECT-SAFE	MT	MT+IVT (bridging treatment)	1	148	1	147	<4.5	parenchymal hemorrhage type 2 associated with an increase of ≥4 points in the NIHSS score or leading to death (SITS-MOST)
TO-ACT	MT + BMT	BMT	1	33	3	34	<24	≥4 points in the NIHSS score or leading to death (SITS-MOST)
Huu An, 2022	MT + IVT	MT	1	30	0	30	<4.5	-
THRACE	MT + IVT	IVT	4	200	3	202	<5	visible intracranial bleeding on CT or MRI plus an increase in the NIHSS score of at least 4 points (SITS-MOST)
DEFUSE 3	MT + BMT	BMT	6	92	4	90	<4.5	an increase of at least 4 points in the NIHSS score that was associated with brain hemorrhage on imaging within 36 hours after symptom onset (SITS-MOST)
IMS 3	MT + IVT	IVT	27	434	13	222	<3	-
EXTEND-IA	MT + IVT	IVT	0	35	2	35	<4.5	parenchymal hematoma type 2 within 36 hours after treatment combined with an increase on the NIHSS of at least 4 points from baseline (SITS-MOST)
SYNTHESIS Expansion	MT	IVT	10	181	10	181	<4.5	-

ESCAPE	MT + IVT	IVT	6	165	4	150	<4.5	-
REVASCAT	MT + IVT	IVT	2	103	2	103	<4.5	parenchymal hemorrhage type 2 on follow-up imaging and neurologic deterioration of at least 4 points on the NIHSS (SITS-MOST)
BASICS	MT	BMT	7	154	1	146	<4.5	Heidelberg Bleeding Classification
MR CLEAN–NO IV	MT	IVT + MT	16	273	14	266	-	Heidelberg Bleeding Classification
BAOCHE	MT + BMT	BMT	6	102	1	88	<8	parenchymal hemorrhage type 2 on follow-up imaging and neurologic deterioration of at least 4 points on the NIHSS (SITS-MOST)
ANGEL-ASPECT	MT + BMT	BMT	14	230	6	225	<24	Heidelberg Bleeding Classification
MR RESCUE	MT	BMT	3	64	2	54	<8	-
TENSION	MT	BMT	7	128	6	125	<6	parenchymal hemorrhage type 2 associated with an increase of ≥4 points in the NIHSS score or leading to death (SITS-MOST)
BEST	MT + BMT	BMT	5	66	0	65	<4.5	intracranial haemorrhage on imaging and an increase of 4 or more points on the NIHSS within 24 h after randomisation
LASTE			15	157	9	157		
TESLA			6	151	2	149		

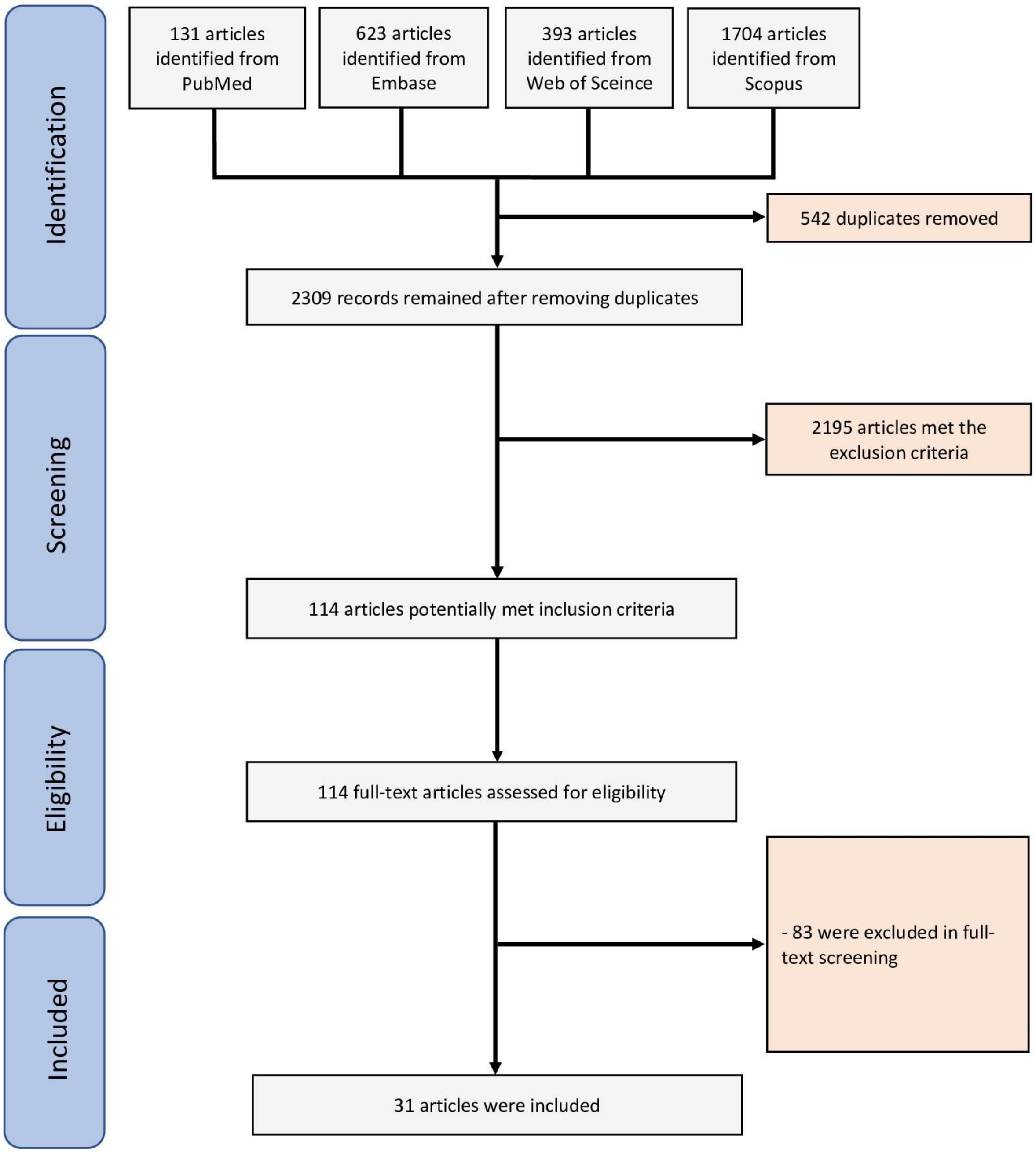
Identification
Screening
Eligibility
Included
131 articles identified from PubMed
623 articles identified from Embase
393 articles identified from Web of Sceince
1704 articles identified from Scopus
542 duplicates removed
2309 records remained after removing duplicates
2195 articles met the exclusion criteria
114 articles potentially met inclusion criteria
114 full-text articles assessed for eligibility
- 83 were excluded in full-text screening
31 articles were included

Supp. Figure 1. PRISMA flow chart.

Risk of bias domains

Study	D1	D2	D3	D4	D5	Overall
ANGEL-ASPECT	+	+	+	+	+	+
ATTENTION	+	+	+	+	+	+
BAOCHE	+	+	+	+	+	+
BASICS	+	+	+	+	+	+
BEST	+	+	+	+	+	+
CHOICE	+	+	+	+	+	+
DAWN	+	+	+	+	+	+
DEFUSE 3	+	+	+	+	+	+
DEVT	+	+	+	+	+	+
DIRECT-MT	+	+	+	+	+	+
DIRECT-SAFE	+	+	+	+	+	+
ESCAPE	+	+	+	+	+	+
EXTEND-IA	+	+	+	+	+	+
Huu An, 2022	X	+	+	+	+	X
IMS 3	+	+	+	+	+	+
LASTE	+	+	+	+	+	+
MR CLEAN–NO IV	+	+	+	+	+	+
MR RESCUE	+	+	+	+	+	+
RESCUE BT	+	+	+	+	+	+
RESCUE-Japan LIMIT	+	+	+	+	+	+
RESILIENT	+	+	+	+	+	+
REVASCAT	+	+	+	+	+	+
SELECT2	+	+	+	+	+	+
SKIP	+	-	+	+	+	-
SWIFT PRIME	+	+	+	+	+	+
SYNTHESIS Expansion	+	+	+	+	+	+
TENSION	+	+	+	+	+	+
THERAPY	+	+	+	+	+	+

Supp. Figure 2. Risk of bias domains for all included trials.

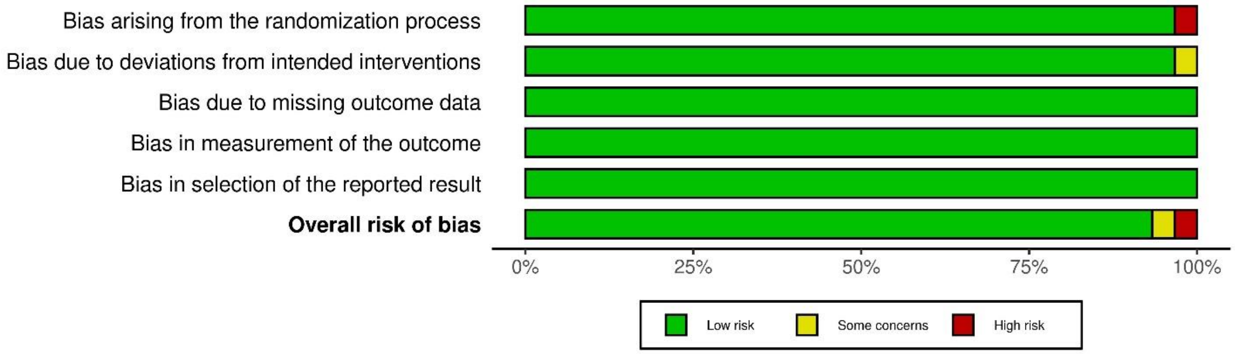

Supp. Figure 3. The overall risk of bias for all trials.

PRISMA CHECKLIST

Section and Topic	Item #	Checklist item	Location where item is reported
**TITLE**			Page 1 (Title on the first page of the manuscript)
Title	1	Identify the report as a systematic review.	Page 1 (Title on the first page of the manuscript).
**ABSTRACT**			
Abstract	2	See the PRISMA 2020 for Abstracts checklist.	
**INTRODUCTION**			
Rationale	3	Describe the rationale for the review in the context of existing knowledge.	Page 2 (Introduction section).
Objectives	4	Provide an explicit statement of the objective(s) or question(s) the review addresses.	Page 2 (Introduction section, where the objectives are outlined).
**METHODS**			
Eligibility criteria	5	Specify the inclusion and exclusion criteria for the review and how studies were grouped for the syntheses.	Page 4 (Methods section, under study selection).
Information sources	6	Specify all databases, registers, websites, organisations, reference lists and other sources searched or consulted to identify studies. Specify the date when each source was last searched or consulted.	Page 4 (Methods section, under search strategy or information sources).
Search strategy	7	Present the full search strategies for all databases, registers and websites, including any filters and limits used.	Page 4 (Methods section, under search strategy).
Selection process	8	Specify the methods used to decide whether a study met the inclusion criteria of the review, including how many reviewers screened each record and each report retrieved, whether they worked independently, and if applicable, details of automation tools used in the process.	Page 4 (Methods section, under study selection).

Section and Topic	Item #	Checklist item	Location where item is reported
Data collection process	9	Specify the methods used to collect data from reports, including how many reviewers collected data from each report, whether they worked independently, any processes for obtaining or confirming data from study investigators, and if applicable, details of automation tools used in the process.	Page 5 (Methods section, under data extraction).
Data items	10a	List and define all outcomes for which data were sought. Specify whether all results that were compatible with each outcome domain in each study were sought (e.g. for all measures, time points, analyses), and if not, the methods used to decide which results to collect.	Page 5 (Methods section, under data items or outcomes).
	10b	List and define all other variables for which data were sought (e.g. participant and intervention characteristics, funding sources). Describe any assumptions made about any missing or unclear information.	Page 6 (Methods section, under risk of bias assessment).
Study risk of bias assessment	11	Specify the methods used to assess risk of bias in the included studies, including details of the tool(s) used, how many reviewers assessed each study and whether they worked independently, and if applicable, details of automation tools used in the process.	Page 6 (Methods section, under statistical analysis).
Effect measures	12	Specify for each outcome the effect measure(s) (e.g. risk ratio, mean difference) used in the synthesis or presentation of results.	Pages 6-7 (Methods section, under data synthesis or statistical analysis).
Synthesis methods	13a	Describe the processes used to decide which studies were eligible for each synthesis (e.g. tabulating the study intervention characteristics and comparing against the planned groups for each synthesis (item #5)).	Pages 6-7 (Methods section, under data synthesis or statistical analysis).
	13b	Describe any methods required to prepare the data for presentation or synthesis, such as handling of missing summary statistics, or data conversions.	Pages 6-7 (Methods section, under data synthesis or statistical analysis).
	13c	Describe any methods used to tabulate or visually display results of individual studies and syntheses.	Pages 6-7 (Methods section, under data synthesis or statistical analysis).
	13d	Describe any methods used to synthesize results and provide a rationale for the choice(s). If meta-analysis was performed, describe the model(s), method(s) to identify the presence and extent of statistical heterogeneity, and software package(s) used.	Pages 6-7 (Methods section, under data synthesis or

Section and Topic	Item #	Checklist item	Location where item is reported
			statistical analysis).
	13e	Describe any methods used to explore possible causes of heterogeneity among study results (e.g. subgroup analysis, meta-regression).	Pages 6-7 (Methods section, under data synthesis or statistical analysis).
	13f	Describe any sensitivity analyses conducted to assess robustness of the synthesized results.	Pages 6-7 (Methods section, under data synthesis or statistical analysis).
Reporting bias assessment	14	Describe any methods used to assess risk of bias due to missing results in a synthesis (arising from reporting biases).	Location: Page 7 (Methods section, under reporting biases).
Certainty assessment	15	Describe any methods used to assess certainty (or confidence) in the body of evidence for an outcome.	Page 7 (Methods section, under certainty of evidence).
**RESULTS**			
Study selection	16a	Describe the results of the search and selection process, from the number of records identified in the search to the number of studies included in the review, ideally using a flow diagram.	Location: Page 8 (Results section, where the number of studies included/excluded is discussed).
	16b	Cite studies that might appear to meet the inclusion criteria, but which were excluded, and explain why they were excluded.	Location: Page 8 (Results section, where the number of studies included/excluded is discussed).
Study characteristics	17	Cite each included study and present its characteristics.	Page 8 (Results section, under study characteristics).
Risk of bias in studies	18	Present assessments of risk of bias for each included study.	Page 8 (Results section, where the

Section and Topic	Item #	Checklist item	Location where item is reported
			risk of bias in included studies is reported).
Results of individual studies	19	For all outcomes, present, for each study: (a) summary statistics for each group (where appropriate) and (b) an effect estimate and its precision (e.g. confidence/credible interval), ideally using structured tables or plots.	Pages 8-9 (Results section, where individual study results are presented).
Results of syntheses	20a	For each synthesis, briefly summarise the characteristics and risk of bias among contributing studies.	Pages 9-10 (Results section, where the synthesis results are reported).
	20b	Present results of all statistical syntheses conducted. If meta-analysis was done, present for each the summary estimate and its precision (e.g. confidence/credible interval) and measures of statistical heterogeneity. If comparing groups, describe the direction of the effect.	Pages 9-10 (Results section, where the synthesis results are reported).
	20c	Present results of all investigations of possible causes of heterogeneity among study results.	Pages 9-10 (Results section, where the synthesis results are reported).
	20d	Present results of all sensitivity analyses conducted to assess the robustness of the synthesized results.	Pages 9-10 (Results section, where the synthesis results are reported).
Reporting biases	21	Present assessments of risk of bias due to missing results (arising from reporting biases) for each synthesis assessed.	Page 10 (Results section, where reporting biases are discussed).
Certainty of evidence	22	Present assessments of certainty (or confidence) in the body of evidence for each outcome assessed.	Pages 10-11 (Results section, under discussion of certainty or confidence in the evidence).
**DISCUSSION**			

Section and Topic	Item #	Checklist item	Location where item is reported
Discussion	23a	Provide a general interpretation of the results in the context of other evidence.	Pages 11-12 (Discussion section).
	23b	Discuss any limitations of the evidence included in the review.	Pages 11-12 (Discussion section).
	23c	Discuss any limitations of the review processes used.	Pages 11-12 (Discussion section).
	23d	Discuss implications of the results for practice, policy, and future research.	Pages 11-12 (Discussion section).
**OTHER INFORMATION**			
Registration and protocol	24a	Provide registration information for the review, including register name and registration number, or state that the review was not registered.	Page 4 (Methods section, under registration information).
	24b	Indicate where the review protocol can be accessed, or state that a protocol was not prepared.	Page 4 (Methods section, under registration information).
	24c	Describe and explain any amendments to information provided at registration or in the protocol.	Page 4 (Methods section, under registration information).
Support	25	Describe sources of financial or non-financial support for the review, and the role of the funders or sponsors in the review.	Page 1 (Funding section).
Competing interests	26	Declare any competing interests of review authors.	Page 1 (Conflict of Interest statement).
Availability of data, code and other materials	27	Report which of the following are publicly available and where they can be found: template data collection forms; data extracted from included studies; data used for all analyses; analytic code; any other materials used in the review.	Page 12 (Data availability or other information).
